# Machine learning in internet financial risk management: A systematic literature review

**DOI:** 10.1371/journal.pone.0300195

**Published:** 2024-04-16

**Authors:** Xu Tian, ZongYi Tian, Saleh F. A. Khatib, Yan Wang

**Affiliations:** 1 Science and Technology Finance Key Laboratory of Hebei Province, Hebei Finance University, Baoding, Hebei, China; 2 Faculty of Management, Universiti Teknologi Malaysia, Johor Baru, Malaysia; 3 Faculty of Management, Hebei Finance University, Baoding, Hebei, China; 4 BoHai College, Hebei Agricultural University, Cangzhou, Hebei, China; Abu Dhabi University, UNITED ARAB EMIRATES

## Abstract

Internet finance has permeated into myriad households, bringing about lifestyle convenience alongside potential risks. Presently, internet finance enterprises are progressively adopting machine learning and other artificial intelligence methods for risk alertness. What is the current status of the application of various machine learning models and algorithms across different institutions? Is there an optimal machine learning algorithm suited for the majority of internet finance platforms and application scenarios? Scholars have embarked on a series of studies addressing these questions; however, the focus predominantly lies in comparing different algorithms within specific platforms and contexts, lacking a comprehensive discourse and summary on the utilization of machine learning in this domain. Thus, based on the data from Web of Science and Scopus databases, this paper conducts a systematic literature review on all aspects of machine learning in internet finance risk in recent years, based on publications trends, geographical distribution, literature focus, machine learning models and algorithms, and evaluations. The research reveals that machine learning, as a nascent technology, whether through basic algorithms or intricate algorithmic combinations, has made significant strides compared to traditional credit scoring methods in predicting accuracy, time efficiency, and robustness in internet finance risk management. Nonetheless, there exist noticeable disparities among different algorithms, and factors such as model structure, sample data, and parameter settings also influence prediction accuracy, although generally, updated algorithms tend to achieve higher accuracy. Consequently, there is no one-size-fits-all approach applicable to all platforms; each platform should enhance its machine learning models and algorithms based on its unique characteristics, data, and the development of AI technology, starting from key evaluation indicators to mitigate internet finance risks.

## 1. Introduction

With the rapid development of internet technology and the arrival of the intelligent era, traditional financial enterprises have gradually expanded their online business operations and are embracing the new format of internet financial services along with the internet financial platform companies that have emerged since 2012 [1]. Internet finance has rapidly developed due to its convenience, real-time nature, and no geographical limitations, resulting in the expansion of market size, number of participants, and services or products offered [2], However, it also faces significant risks, as evidenced by the large number of problems with P2P platforms in 2018. Compared with traditional financial services, internet finance has relatively low barriers to entry, smaller amounts, faster speeds, and more relaxed audits, which has led to higher requirements for credit risk control, fraud prediction, and other risk prevention measures in internet financial platforms [3, 4]. Research related to risk identification, risk alert, and risk supervision based on big data [5–8], blockchain [9, 10], artificial intelligence [4, 11, 12] and machine learning algorithms [1, 2] is progressively unfolding.

The internet finance refers to a business model wherein traditional financial institutions or internet companies utilize internet technology to provide financial-related services such as financing, payment, investment, and information intermediation on the internet [13]. Over a span of two years starting from 2016, more than 200 internet finance companies in China alone faced instances of default, involving issues like borrower delinquency, platform fraud, and cyberattacks [2]. Only in 2018, the thriving P2P internet finance platforms in China plummeted from 6385 to 1595 by August, resulting in significant losses for investors [14]. Internet financial services offer rapid response times, thereby enhancing user satisfaction. Consequently, swift identification of potential risks is crucial [15, 16]. Considering that the internet will remain a pivotal direction for the development of the financial industry for the foreseeable future, with an increasing number of services offered by major financial institutions, such as banks, being conducted through online channels, this paper focuses on the issue of financial risk prevention in the internet domain. Research on internet financial risk warning can effectively nip potential risks in the bud [17], as traditional credit scoring card models can no longer cater to the needs of business development and security balance [2]. The aim is to explore how different machine learning methods can better identify and mitigate internet finance risks, particularly when traditional credit rating methods are not well-suited for the rapid and efficient nature of the internet. This paper adopts a systematic literature review approach to examine the various machine learning models and algorithms utilized by different scholars in assessing internet finance risks. This comprehensive review aims to gain insights into the application of machine learning algorithms in this field and the outcomes across different contexts, thereby comparing the suitability of different algorithms in this domain.

The significance and main contributions of this paper are manifested in several aspects. Firstly, it innovatively employs a systematic literature review approach to delineate the landscape of machine learning models and algorithms in internet finance risk management. Through a systematic analysis of previous research achievements, this study comprehensively reviews and compares the approaches and outcomes of machine learning in internet financial risk warning and identification. Secondly, while traditional credit scoring methods and various machine learning algorithms are commonly used in risk management for internet finance platforms, previous literature has compared these methods in different contexts. This paper provides a clear and comprehensive classification and summary analysis of the application of these methods in internet finance platforms. Thirdly, building upon the existing landscape, we believe this paper provides a clear roadmap for future research on this topic, outlining research directions and themes to bridge knowledge gaps. Fourthly, from a practical standpoint, the various frameworks and methods for internet financial risk identification provided by this study can assist internet financial companies in identifying their weaknesses and enhancing risk prevention measures. This, in turn, can elevate their service quality, facilitating more widespread and stable financial services.

The subsequent structure of this study is outlined as follows. Section 2 presents the literature review; Section 3 introduces the methods and strategies of this paper; Section 4 shows the results; Section 5 discusses the findings; Section 6 presents the conclusions and the last section is the future research suggestions.

Due to scholars’ utilization of various data sets and scenarios in their research, coupled with the rapid development of machine learning model algorithms, including large models like Transformer, which currently lack research literature on internet finance risk, this paper cannot provide a unified conclusion. Instead, practitioners could select models and algorithms that best suit their own circumstances and data based on the evaluative findings presented in this paper.

## 2. Literature review

Scholars have proposed the utilization of machine learning techniques [14, 18, 19] to predict credit risks by collecting and mining internet data. This approach has yielded superior predictive outcomes compared to conventional methods. Even within the same data sources, machine learning models exhibit greater accuracy [2, 8], stability [8], predictive precision [19, 20], and efficiency [20] in contrast to traditional credit scoring models.

Mirza et al. [19] compared various methods such as Naïve Bayes, Random Forest, and DLNN, and computed the accuracy of different models, revealing an enhancement in the precision of internet finance credit detection and prediction. However, researchers have discovered variations in efficiency and outcomes among different machine learning models and algorithms. Thus, developing superior algorithms and more efficient, reliable machine learning models for internet financial risk prediction has become an urgent challenge to address.

The research on the topic of internet financial risk has a long history [21], encompassing both quantitative empirical analyses [22] and qualitative descriptions [1], as well as comprehensive review studies [13]. There are analyses employing quantitative platform data [2, 14] and those conducted using textual data [23, 24]. Studies have delved into various subtopics such as risk perception [22], risk identification [24], and risk regulation [12], rendering the research on internet financial risk quite extensive.

However, the exploration of internet financial risk from the perspective of machine learning models emerged relatively late. The application of this approach to internet financial risk warning and risk management research began as early as 2019 [15], gradually gaining momentum alongside technological development [11, 19, 25]. The primary focus of these studies lies in the selection of model methodologies [17, 20, 26] and the construction of risk systems [1, 27, 28]. However, to date, there has been no comprehensive review article or study systematically outlining the state of this emerging yet critical research field. This is precisely the contribution of the present study.

The primary object of this study is to elucidate the application and research status of various machine learning algorithms or models in identifying and warning about internet financial risks. Using a systematic literature review approach, a comprehensive analysis of relevant literature in this field is conducted. Currently, there are only a limited number of articles on this topic [11, 25, 27], and our study addresses the following three main questions through analysis, clarifying the current state of research advancement and literature gaps in this field, as well as the differences between various internet financial risk identification and warning methods.

Q1. What machine learning algorithms have been studied in the literature for internet financial risk identification and warning, and have these algorithms and models all shown improvement?
Q2. How is the application status of the aforementioned algorithms and models?
Q3. Is there a best-suited machine learning algorithm for most internet financial platforms?

In this study, a systematic literature review method is employed to investigate the above questions. This method is well-suited for concentrating on a specific topic, providing a panoramic view, offering a more comprehensive understanding of the chosen domain, and highlighting gaps and future research directions [29–31].

## 3. Methodology

Following the standardized Systematic Literature Review (SLR) [32, 33], this study advanced its research. Initially, we opted for the Scopus and Web of Science (WOS) databases as sources, conducting searches for all publications related to "internet financial risk" across various years. Scopus, being the world’s largest abstract and citation database, provides an extensive repository of abstracts and citations. Web of Science, on the other hand, is a comprehensive, multidisciplinary, core journal citation indexing database. Both databases are globally authoritative and specialized platforms for data retrieval, offering advanced search functionalities. This facilitates our ability to obtain relevant search results quickly, efficiently, and comprehensively.

### 3.1 Sample identification

In this study, we employed a keyword-based literature retrieval strategy [29, 34]. To gather all relevant literature and research, we formulated multiple search strings related to "internet financial risk". Considering the diverse expressions in English, where internet financial could also be referred to as "online finance," "network finance," or "Fintech," we compiled all potentially involved keywords listed in Table 1 and combined them through permutations using the Boolean operator "or". Furthermore, recognizing variations in the usage of terms like "finance" and "financial," we used the asterisk "*" to represent inconsistent parts, aiming to comprehensively cover the complete continuum of the phrase "internet financial risk". In the Scopus database, we employed the search method of "Title-Abstract-Keywords". In the Web of Science database, we used "Topic" as the search mode, and we narrowed down the search scope to three major citation databases: the Science Citation Index (SCI), Social Sciences Citation Index (SSCI), and Arts & Humanities Citation Index (A&HCI), to ensure the quality of the source journals. The final search strings are as presented in Table 1. The search date for all the data is August 9, 2023, and all literature cited in this study is up to that date.

**Table 1:** Keywords and searching query string.

	“Internet” terms	“Finance” terms	“Risk” terms
Keywords	internet	finance	risk
	online	financial	venture
	network	bank	threat
	net	banking	danger
	fintech		safe
			safety
			supervise
			supervision
Search query string	("internet* financ* risk") or ("internet* financ* threat") or ("internet* financ* venture") or ("internet* financ* danger") or ("internet financ* safe*") or ("Internet financ* supervis*") or ("online financ* safe*") or ("online financ* supervis*") or ("online financ* danger") or ("online financ* venture") or ("online financ* threat") or ("online financ* risk") or ("internet bank* risk") or ("internet bank* venture") or ("internet bank* threat") or ("internet bank* danger") or ("internet bank* supervis*") or ("internet bank* safe*") or ("fintech* risk") or ("fintech* safe*") or ("fintech* supervis*") or ("fintech* venture") or ("fintech* danger") or ("fintech* threat") or ("online bank* risk") or ("online bank* venture") or ("online bank* danger") or ("online bank* threat") or ("online bank* safe*") or ("online bank* supervis*") or ("network financ* risk") or ("network financ* threat") or ("network financ* venture") or ("network financ* danger") or ("network financ* safe*") or ("network financ* supervis*")		

### 3.2 Inclusion and exclusion criteria

Following the search using the aforementioned keyword strings, the initial results in the Scopus and Web of Science databases were 116 and 48 publications, respectively. After following the approach of Khatib et al. [31] and Khatib et al. [32], we refined the results by limiting the language to "English", reducing the counts to 113 and 48. Further refining to "journal articles" and resulted in 70 and 48 publications. Subsequently, in the Scopus database, we narrowed down the "Subject area" to categories including "Computer Science", "Economics, Econometrics and Finance", "Business, Management and Accounting", "Engineering", "Mathematics", "Social Sciences", "Decision Sciences" and "Multidisciplinary". In the WOS database, we limited the "research area" to "Business Economics", "Computer Science", "Mathematics", "Telecommunications", "Engineering", "Operations Research Management Science", "Environmental Sciences Ecology" and "Science Technology Other Topics", yielding 68 and 48 publications respectively.

Then we merged the above-mentioned literature while removing duplicates, resulting in 70 articles. Subsequently, we conducted title screening and excluded 7 articles. The remaining 63 publications were subjected to abstract reading and screening, yielding 47 relevant articles. Finally, we thoroughly read these remaining publications, retaining those that incorporated concepts related to machine learning and eliminating others unrelated to the subject. We have also excluded a paper that has been retracted. This led to the final selection of 17 literatures focusing on the application of machine learning for internet financial risk identification and warning.

**Fig 1:**
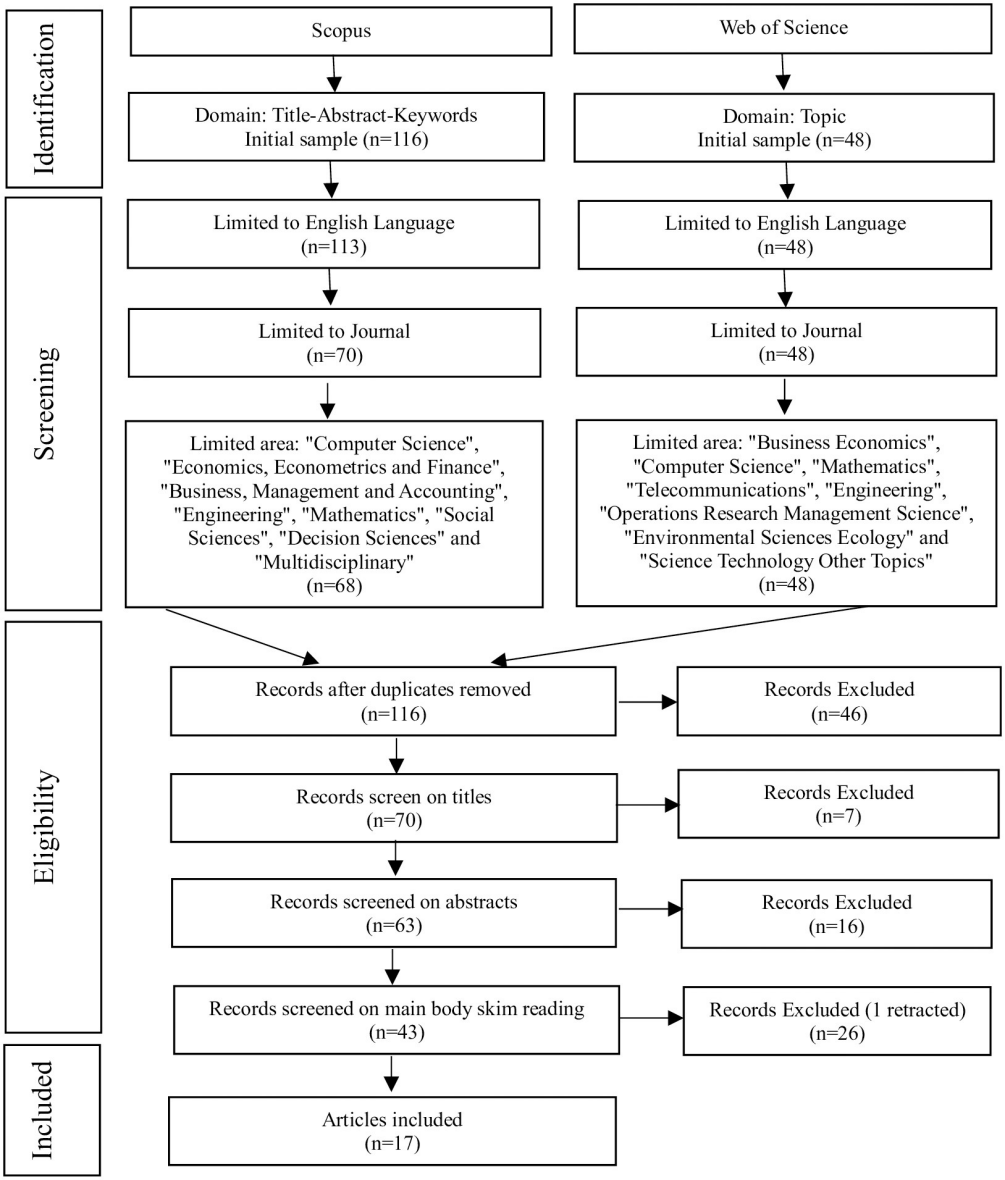
The process of searching the literature.

Fig 1 illustrates the process conducted in this study, encompassing database searches, refinement, merging, deduplication, screening, and eligibility selection. Unlike existing articles that solely focus on "internet finance risk" [22], "financial technology" [35], or "credit risk" [13], this study employs a systematic review approach to concentrate on the application and exploration of various machine learning methodologies in the realm of "internet financial risk." Despite the limited number of publications, this review comprehensively assesses and evaluates literature in this field. It not only analyzes numerous models and algorithms applied in the domain of internet financial risk but also systematically examines aspects like annual publication trends, regional publication trends, relevant research methods, evaluation metrics, and more.

For the aforementioned literature, this paper will focus on examining the machine learning models employed by scholars in the field of internet finance risk management, as well as how these models perform across different platforms and scenarios. Therefore, we will compare the applicability of different models and algorithms in this field based on the development history, application domains, and advantages of machine learning. The results will be presented in Section 4.5.

When evaluating and assessing model performance, the goal is to ensure that the model correctly classifies samples, meaning that the actual situation of the sample data matches the model’s predictions as closely as possible. Therefore, for binary classification problems, there are four different scenarios:

1. The model predicts positive, and the actual situation is also positive, indicating that the model prediction is true, known as the True Positive (TP) scenario.
2. The model predicts negative, but the actual situation is positive, indicating that the model prediction is false, known as the False Negatives (FN) scenario.
3. The model predicts positive, but the actual situation is negative, indicating that the model prediction is false, known as the False Positives (FP) scenario.
4. The model predicts negative, and the actual situation is also negative, indicating that the model prediction is true, known as the True Negatives (TN) scenario.

TP, FN, FP, and TN respectively represent the sample counts for the four scenarios described above. Therefore, machine learning model assessment is based on these four scenarios, and a series of metrics have been developed to judge the model’s performance. This paper will present the results and explanations based on the main metrics applied in the literature in the "Results" section.

Despite including data from WOS and Scopus, there is still a possibility of not encompassing all relevant literature. However, considering the authority of the literature research, this paper still relies on the two aforementioned databases, which are of higher quality and more authoritative in content.

## 4. Results

### 4.1 Publication trends

**Fig 2:**
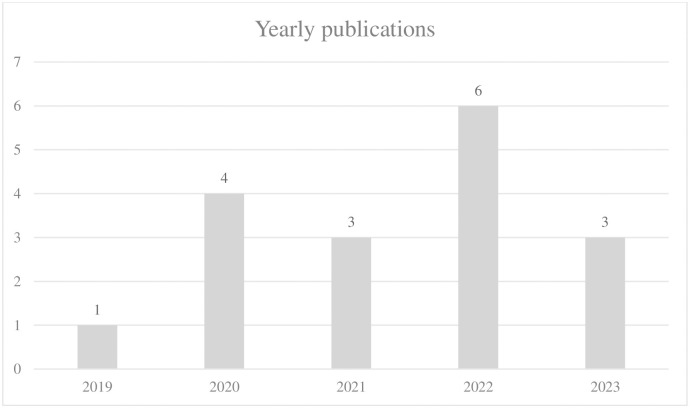
Yearly publications volume about this theme.

The popularization of Internet financial services occurred around 2010, while research focusing on Internet financial risks began in 2012 [21]. Thanks to a plethora of algorithmic innovations in the field of computer algorithms, machine learning, deep learning, and other methods have gradually been applied to Internet financial risk analysis. This has led to a growing interest in the subject. In our sample literature, the earliest document on this topic dates back to 2019 which was conducted by Noor et al. [15], with only one publication. Subsequently, the number of publications started to increase gradually, reaching 6 by 2022. As of August 2023, there have been three more publications, indicating a relatively limited volume overall. This suggests that research on the application of these specific methods in this particular field is still relatively insufficient. The yearly publications volume shown in (Fig 2).

### 4.2 Geographical distribution

As shown in Table 2, this section presents the annual regional distribution of all the references in this paper. It’s quite evident that out of the 17 documents, 11 of them are based on Chinese Internet financial data [14, 27]. Chinese scholars or researchers using Chinese data for machine learning algorithms in Internet financial risk analysis stand out as the driving force behind research on this topic. The sources of all this data primarily fall into three categories: national-level data [26], data from Internet financial platforms or related enterprises [17, 20], and individual lending data from platforms [2, 14], all of which are also detailed in Table 2. Regarding this subject, there are studies focused on Europe, the United States, and those utilizing global Internet financial platform data. Additionally, three articles do not precisely specify the regional focus of their research [7, 25, 36].

**Table 2:** The annual reginal distribution.

	2019	2020	2021	2022	2023	Total
China		3	1	5	2	11
*companies*			*1*	*3*	*1*	*5*
*individual*		*3*		*1*	*1*	*5*
*country*				*1*		*1*
Europe		1				1
USA					1	1
Global	1					1
Not mention			2	1		3

This phenomenon might be attributed to the fact that in China, after a period of rapid and unchecked growth of Internet financial platforms [17], serious risk issues emerged, involving numerous defaults, platform escape with money, and other problems [23], the number of platform drop to 1/4 from the top year [14]. Although Internet financial is an emerging financial service model, it has not altered the fundamental nature of financial services. Risk prevention remains a crucial and central aspect [27, 28]. Consequently, Chinese scholars and professionals in the financial industry have shown a great deal of concern about Internet financial risk. They aim to utilize various methods to mitigate these risks, promote the healthy development of the industry and Internet financial services, thus generating a heightened demand [8].

### 4.3 Literature focus

Upon reviewing all the literatures, it becomes evident that these documents broadly focus on two distinct core aspects. One category of literature primarily revolves around comparison. These papers compare the differences in final risk identification, risk prediction, and risk supervision using various algorithms or models [11, 19, 20]. The objective is to identify the most suitable approach for applying sample data, thereby better assisting platform companies or other entities in mitigating Internet financial risks. A total of 14 documents fall into this category. The other category of literature centers on designing or innovating Internet financial risk systems, applying relevant data to construct appropriate risk identification or risk prediction systems [27, 28, 36]. Although these two categories of literature emphasize slightly different core points, their ultimate goals are risk reduction and enhancing operational stability. Both categories utilize machine learning-related models or algorithms, leading to a convergence of approaches. This underscores the diverse perspectives and research angles in understanding the practical applications of computer technology in the realm of Internet financial risk. As shown in Table 3.

**Table 3:** Literature research focus on and risk fields.

	2019	2020	2021	2022	2023	Total
Construction system		1	1	2	2	6
Method selection	1	4	2	5	2	14
Risks of Internet financial platforms		1	1	4	2	8
Credit risk assessment and early warning		3	2			5
Internet financial market risk				2		2
Fraud Detection					1	1
Cyber threat					1	1

Currently, there are numerous sources of risk in internet finance, and the application of machine learning in internet finance risk management covers a wide range of areas and directions. From the literature reviewed, machine learning is primarily applied in the following five different types of risk management:

1. Internet financial platforms risk: This category focuses on analyzing and alerting various risks that may occur during the operation and management processes of internet finance platforms using different machine learning algorithms [7, 23, 28]. For instance, Feng and Qu [18] designed an RBF neural network model optimized by genetic algorithms and established an evaluation index system for internet finance risk. Han et al. [8] decomposed it into four major components: credit risk, liquidity risk, interest rate risk, and technology risk.
2. Credit risk assessment and early warning: This area primarily studies the early identification and prediction of borrower credit using various machine learning algorithms. It is believed that suitable machine learning algorithms can effectively promote the identification of credit risks in lending, leading to higher predictive accuracy [2, 11, 14, 25, 36].
3. Internet financial market risk: This category focuses on identifying and analyzing risks in the internet finance market to enhance the level of internet finance risk management [18, 27].
4. Fraud Detection: This involves analyzing the efficacy of machine learning models in fraud detection, aiming to identify danger signals in economic datasets to detect future fraudulent activities [19].
5. Cyber threat: This area explores how machine learning models and algorithms can identify advanced network attack patterns and conduct automated network threat attribution analysis and prediction [15]. The distribution of different risk types in the literature is shown in Table 3.

### 4.4 Fields of sciences

**Fig 3:**
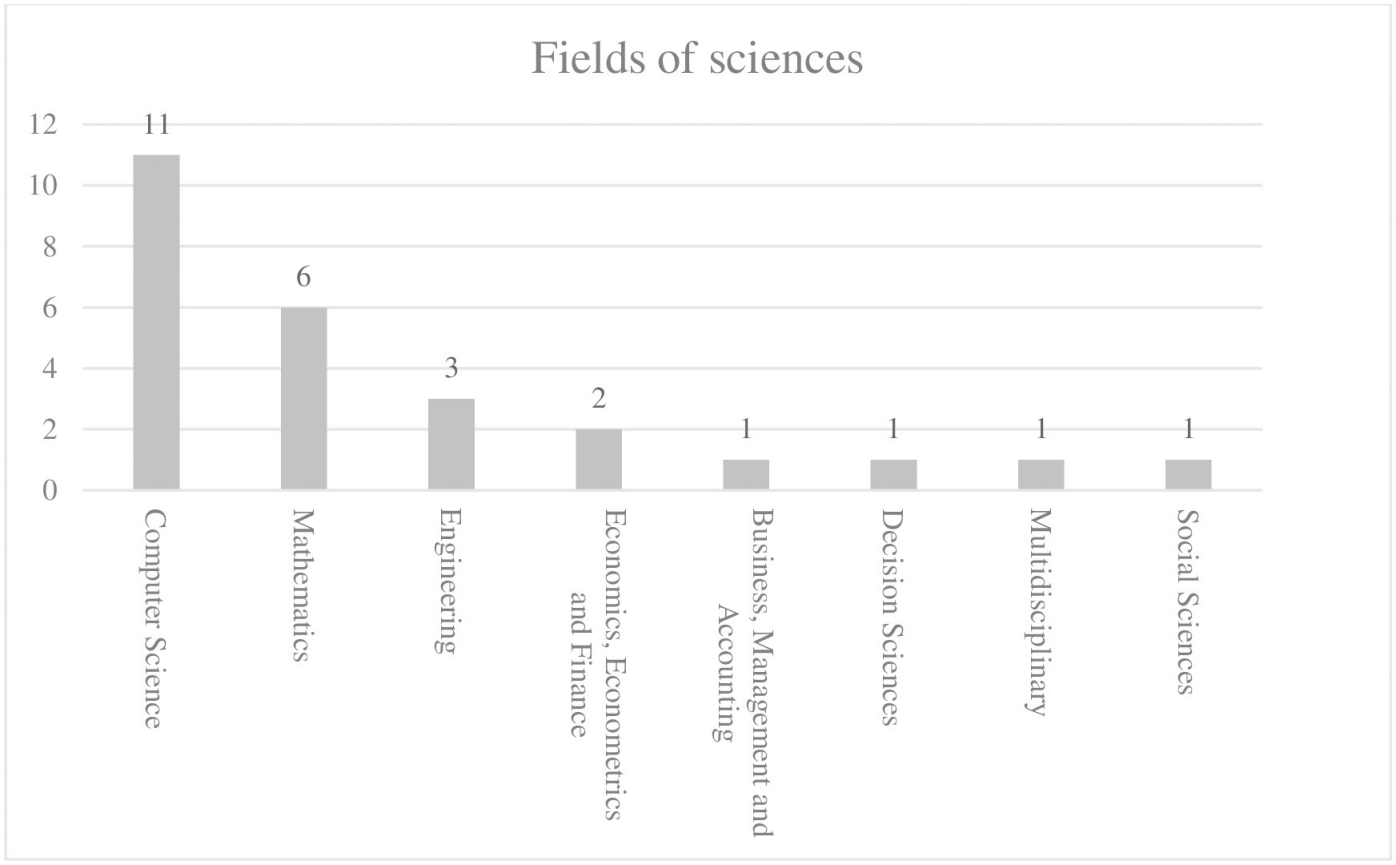
Fields of sciences registered for literatures.

Fig 3 provides a detailed overview of the science subject areas in which the articles from the Scopus database are classified. According to the categorization method of the Scopus database, all the literature has been divided into a total of eight different subject areas. The highest number of papers falls under "Computer Science," followed by "Mathematics" and "Engineering," with no more than two papers in any other category. This indicates that although the theme of "Internet financial risk" leans more toward the field of economics and management, the literature predominantly focuses on the methodological aspects of risk identification and prediction. This aligns with the content discussed in the previous section.

### 4.5 Machine learning methods

Based on the machine learning methods employed in the literature covered in this paper, they can be broadly categorized into five types: Traditional Machine Learning Algorithms, Deep Learning and Neural Networks, Optimization Algorithms, Data Preprocessing and Enhancement, and Other Methods. In the following sections, we will categorically discuss the methods utilized in the literature. Table 4 presents the annual distribution of all methods used in the sample literature. It should be noted that the same method can be classified into different categories based on various classification approaches. The above classification is solely aimed at facilitating the organization and expression of the literature content.

**Table 4:** Annual statistical table of machine learning methods and classification.

Category	Subclass	Name	2019	2020	2021	2022	2023	Total	Sum
Traditional Machine Learning Algorithms	Classification Algorithm	Logistic Model		2	2	1	1	6	30
		Random Forest	1		1		2	4	
		Gaussian Naïve Bayes Model	1		1		2	4	
		Decision Tree	1		1		2	4	
		K-Nearest Neighbor (KNN)	1		1		1	3	
		SVM Model		1	1	1		3	
	Association Analysis and Clustering	K-means		1		1		2	
		Apriori Algorithm				1		1	
	Data Dimension Reduction and Enhancement	Traditional RBF-NN				2		2	
		Complementary Neural Network (CMTNN)				1		1	
Deep Learning and Neural Networks	Basic Neural Network	BP Neural Network (BPNN)			1	4	2	7	25
		Deep Learning Neural Network (DLNN)	1			1	2	4	
	Convolutional Neural Network	Convolutional Neural Network (CNN)		1			1	2	
	Recurrent Neural Network	Long- and Short-Term Memory (LSTM)		1			1	2	
		Recurrent Neural Network (RNN)		1				1	
		Bi-directional Long Short-Term Memory (BiLSTM)		1				1	
	Integration Model	XGBoost Model		2			1	3	
		AdaBoost Algorithm					1	1	
		General Regression Neural Network (GR-NN)				1		1	
		Deep & Cross Network (DCN)					1	1	
	Other Neural Networks	Restricted Boltzmann Machines (RBMs)					1	1	
		Probabilistic-Neural Network(PNN)				1		1	
Optimization Algorithms	Optimization Algorithms	Genetic Algorithm (GA)				3		3	6
		GABP Algorithm				1		1	
		GABP Algorithm Based on Simulated Annealing Optimization				1		1	
		ACO-Optimized RBF Algorithm				1		1	
Data Preprocessing and Enhancement	Data Balancing and Enhancement	Synthetic Minority Over-sampling Technique Algorithm(SMOTE)		1				1	5
	Feature Processing and Selection	Group Method of Data Handling (GMDH)		2				2	
		Weight of Evidence(WOE)			1	1		2	
Others	Text Processing and Entity Recognition	Named Entity Recognition (NER)		1				1	4
	Others	Fuzzy Analytic Hierarchy Process (FAHP)				1		1	
		Big Data				1		1	
		Internet of Things(IoT)				1		1	

#### 4.5.1 Traditional machine learning algorithms

Among all the literature, traditional machine learning methods are mentioned and utilized a total of 30 times, making it the most frequently used category among the four mentioned above. This suggests that methods introduced or adopted earlier have a higher frequency of use in the context of internet financial risk, implying their relatively mature applicability. Among these, the most commonly used method in the literature is the Logistic Model, appearing in 6 articles, followed by the Random Forest, Gaussian Naïve Bayes Model and Decision Tree methods are each used in 4 articles. It’s worth noting that among all the traditional machine learning methods, the more frequently used methods belong to the category of Classification Algorithms.

Due to its strong interpretability, the Logistic Model is the most frequently utilized model in credit scoring [2, 36]. Since the Logistic Model’s predictions can output the probability of belonging to a certain category for a record [25], adopting methods like the logistic model reveals that machine learning models have an advantage in identifying key influencing factors affecting credit customer default performance. Bussmann et al. [11] and Wu et al. [1] have also compared the logistic model with other machine learning models. Scholars utilized data from European Credit Assessment Institutions (ECAIs) focusing on commercial loans for small and medium-sized enterprises (SMEs) obtained from P2P platforms to construct a logistic regression scoring model. This model incorporated financial data on assets and liabilities, as well as network centrality indicators derived from similarity networks, to estimate the default probability of each company. A comparison was made with the results of models employing the XGBoost tree algorithm, and it was found that for internet financial risk, newer deep learning methods generally exhibit higher predictive accuracy [11].

The Random Forest method is based on the decision tree approach, where each branch of the decision tree represents a potential decision, event, or response [15]. Decision trees can achieve very low bias, but they also exhibit strong instability and sensitivity [25]. Therefore, the Random Forest method employs random data sampling and replacement strategies to construct decision trees, mitigating the issue of inconsistent sample selection due to varying tree shapes [19]. When using the Random Forest method for risk stratification on internet financial platforms, its objective is to reduce variance. This method is applicable to machine learning tasks involving classification and regression, offering higher accuracy and robustness [19]. Compared to other machine learning methods, it yields more robust and accurate results [1, 25]. For internet financial platforms, greater accuracy and robustness are crucial for identifying risks with greater precision and reliability. Therefore, the Random Forest method is widely applied across various platforms.

Due to the relatively simpler implementation of Naive Bayes models and their requirement of smaller training data, they are capable of handling both continuous and discrete data. Naive Bayes is a probabilistic classifier based on the principles of conditional probability in Bayes theorem [15]. They also offer rapid prediction capabilities, making them particularly suitable for real-time forecasting [15]. Furthermore, they can conduct sentiment analysis and scoring of online user information, effectively evaluating user eligibility [1]. Additionally, Bayes models exhibit a higher level of accuracy [19, 25]. K-Nearest Neighbor (KNN) is a supervised machine learning algorithm that doesn’t require prior knowledge and can classify based on the majority vote of its neighbors. It’s particularly well-suited for large-scale financial service platforms [15, 17]. The KNN method can be employed in conjunction with other techniques to obtain the fitness value. However, in the study by Mirza et al. [19], KNN was found to have the lowest accuracy among the five methods employed.

Of course, there are also other methods like traditional RBF-NN [18, 27], and complementary-neural network (CMTNN) [27] applied in the existing literature as innovative models and approaches for internet financial risk prediction. Overall, these methods have the potential to enhance the accuracy and speed of traditional predictions. Consequently, models like the Logistic model, Bayes model, and Random Forest, have become more mature in the field of machine recognition [1, 7], and have found wide application. However, from scholars’ perspectives, newer deep learning and reinforcement learning methods have shown superior performance on specific datasets compared to traditional machine learning algorithms [2, 19]. These methods may find broader applications in the future in the field of internet financial risk identification and early warning.

#### 4.5.2 Deep learning and neural networks

Firstly, in terms of overall quantity, applications related to deep learning and neural network methods in the context of internet financial risk have appeared a total of 25 times, which is equal to the count of applications of traditional machine learning methods. Additionally, from a temporal perspective, deep learning and neural network models had only one literature in 2019, and the combined occurrences in 2020 and 2021 were merely 8. However, since 2022, the frequency has escalated to 17 occurrences, surpassing more than 2 times the occurrences in the preceding three years. Specifically, in 2022 alone, there were 8 occurrences, and by August 2023, there were already 9 instances, signifying a gradual and increasing integration and utilization of deep learning and neural network-related models in the domain of internet financial risk management. Turning to the specifics of method applications, the most utilized is the BP neural network, referenced and employed in a total of 7 literature sources. Following this, there are 4 instances mentioning the Deep Learning Neural Network (DLNN), and subsequently, for the XGBoost Model, Convolutional Neural Network (CNN) and Long- and Short-Term Memory (LSTM), each mentioned in 3, 2, and 2 literature sources, respectively.

In the analysis of internet financial risk, the Backpropagation (BP) neural network stands out as the most frequently applied method across all literature sources. Typically, a BP neural network comprises at least three layers: the input layer, hidden layer, and output layer [8]. This approach does not require a predefined mathematical expression between the input and output layers [25]. Its principle is rooted in the error backpropagation algorithm of a multi-layer feedback network, which involves adjusting thresholds and weights based on the error of results [20, 26]. As a result, the structure of the BP neural network is simpler, while its predictive accuracy and nonlinear processing capabilities are stronger [18, 20]. In the context of analyzing internet financial platform risk management issues, this approach has been widely adopted by scholars [1, 18, 27]. The study utilized data from 65 publicly listed Chinese companies to train optimized neural networks. Testing was conducted using big data from internet finance enterprises spanning from 2015 to 2018, with a comparison drawn against the actual development of the internet finance sector., it has been observed that compared to other models, although the BP neural network yields higher predictive accuracy, it requires the longest training time [18]. Therefore, as a foundational deep learning and neural network method, when combined with other algorithms in subsequent steps, it can produce improved outcomes [18].

In the literature on deep learning for internet financial risk, it is mentioned that the foundation of deep learning operates akin to the neural network systems in the human brain [15], capable of learning from unlabeled or unstructured data. It fundamentally follows a supervised learning approach, enabling a better understanding of the mapping relationship between x and y [26, 37]. Thanks to significant advancements in algorithms and hardware, deep learning can leverage increased levels and neuron counts for modeling, thus making it feasible for application in internet financial risk management [1]. Mirza et al. [19] constructed a database spanning 10 years, comprising 95 companies, using KBW and Nasdaq Financial Technology Rankings, as well as the Nasdaq Insurance (IXIS) Index. The aforementioned data was then used to compare five algorithms, including Naive Bayes, KNN, Decision Tree, Random Forest, and DLNN and found that, in comparison to traditional machine learning methods, the accuracy of deep learning (DLNN) is the highest among all five methods. Scholars have been consistently combining foundational deep learning models with other algorithms in an attempt to explore more suitable deep learning algorithms.

The highly renowned XGBoost optimization model is also rooted in the decision tree algorithm, essentially utilizing the gradient boosting ensemble technique to combine multiple decision tree models [2]. Leveraging gradient descent methods to minimize errors [11], inappropriate trees are pruned, resulting in a high-accuracy gradient tree boosting model [19]. This uniqueness positions the XGBoost Model with a distinctive advantage in handling sparse data. Fan et al. [2] selected a P2P online lending platform in China as the research subject and utilized data from 30,225 short-term loans issued by the platform from August to December 2018. Logistic regression, GMDH, SVM, and XGBoost algorithms were compared for internet finance risk assessment. It was found that the XGBoost model achieved the highest overall accuracy, with a testing set accuracy of 90.1%. Similar conclusions were also drawn in the study by Bussmann et al. [11].

Convolutional Neural Networks (CNN), built upon the foundation of deep learning (DLNN), incorporate convolutional layers designed for data feature extraction [23]. These extracted features are then passed to different network nodes, allowing for layered representation and data learning, ensuring efficient learning processes. CNNs are characterized by sparse connections and weight sharing [23], and have been attempted for prediction tasks, demonstrating performance on par with human experts [19].

Scholars have employed Long Short-Term Memory (LSTM) for researching internet financial risk [19]. LSTM, a specialized Recurrent Neural Network, comprises three control units: input gate, output gate, and forget gate, enabling it to address the challenge of long sequence dependencies in neural networks [23]. Consequently, this enhances the predictive accuracy for high-risk groups in internet finance. Xia et al. [23] improved classification outcomes by incorporating an attention mechanism, and further elevated accuracy by introducing Bi-directional Long Short-Term Memory (BiLSTM) with reverse sequence information using 42,590 Q&A pairs text. This is because BiLSTM consists of both positive and negative LSTMs, enabling a thorough consideration of the contextual information’s influence on the current output. This facilitates the learning of more accurate semantic representations of text, leading to a more comprehensive understanding of its semantics [38]. The consideration of contextual information in the output led to even higher recognition accuracy.

Adaptive Boosting (AdaBoost) involves combining outputs from various methods to enhance classification performance, ensuring a reduction in overall classifier error after each iteration. As a result, this method has achieved high accuracy in internet financial risk models [19]. Methods such as Probabilistic-Neural Network (PNN) [27], general regression neural network (GR-NN) [27], and Restricted Boltzmann Machines (RBMs) can also accelerate the learning process, improving optimization efficiency [1]. By employing various algorithms based on deep learning and neural networks in internet financial risk management, scholars generally find that improvements in machine learning algorithms lead to enhanced accuracy, robustness on validation sets, and even reduced response times. Hence, it can be said that with the aid of more applicable machine learning algorithms, the capability of internet financial risk management is continuously improving, and this improvement process remains ongoing.

#### 4.5.3 Optimization algorithms

The literature also enumerates some optimization algorithms, with the most prominent being Genetic Algorithms(GA) and their enhanced variants based on genetic algorithms [20]. The Genetic Algorithm (GA) is a global optimization algorithm based on probabilistic optimization [20], known for its strong global search capabilities and wide adaptability [18, 23]. The ACO-optimized RBF algorithm possesses high spatial mapping and generalization capabilities [18]. The GABP neural network adopts a distributed storage structure. It demonstrates fast iteration speed, accurate results, good redundancy, and robustness in financial risk identification [20].

Combining the aforementioned Genetic Algorithm and Simulated Annealing Algorithm, the GABP Algorithm Based on Simulated Annealing Optimization method was used, and it was found to have higher accuracy and predictive speed compared to BP neural networks and GABP networks. Guang et al. [20] selected 36 internet finance companies as samples and grouped them based on financial conditions for optimization using GA, GABP, and SA-GABP algorithms. They found that leveraging the global optimization capabilities of various genetic algorithms and applying the optimized networks to predict internet finance risks resulted in favorable prediction outcomes. This provides a scientific basis for credit decision-making and risk prevention in internet finance and banking.

#### 4.5.4 Data preprocessing and enhancement

This section primarily concerns data preprocessing and selection, encompassing three main methods: Synthetic Minority Over-sampling Technique Algorithm (SMOTE), Group Method of Data Handling (GMDH), and Weight of Evidence (WOE), with a total of only 5 applications. The SMOTE algorithm, based on synthetic sample synthesis, enhances data discriminability accuracy by generating new synthetic samples to form a new dataset [2]. The Group Method of Data Handling (GMDH) is a technique to extract significant information from vast and complex data, thereby improving analytical efficiency [14]. This is crucial for handling the substantial and intricate data inherent to open attributes in internet financial platforms. In the research by Fan et al. [2], GMDH achieved accuracy second only to XGBoost. Weight of Evidence (WOE) is primarily employed to assess the relationship between features and targets, examining default situations in internet financial platforms [7, 36]. Through appropriate data preprocessing, feature selection, and effective algorithm integration, this serves as a pivotal step in ensuring accurate risk assessment for internet financial platforms.

#### 4.5.5 Other methods

Other methods mentioned in the literature include Named Entity Recognition (NER), which primarily involves text processing and entity identification. NER falls within the domain of text processing and natural language processing techniques and can identify names, specific locations, and other contextually significant content within text [23]. There is also the Fuzzy Analytic Hierarchy Process (FAHP), an analytical method used for multi-criteria decision-making problems [28], and methods related to big data and the Internet of Things (IoT) [27]. While not the main focus here, it’s evident that these methods, particularly NER in conjunction with emotional analysis, can effectively broaden the applicability of machine learning in internet financial risk identification.

### 4.6 Literature findings

Table 5 presents the titles and research findings of selected literature, aiming to comprehend the overall research conclusions, current status, and trends of this issue. This provides potential research directions for future studies. Based on the aforementioned analysis and the research findings listed in Table 5. (1). It can be established that internet financial risk is a widely recognized and crucial latent issue. Machine learning, as a novel computational technology, whether through foundational algorithms or complex algorithm combinations, offers significant advancements in risk prevention compared to traditional credit scoring methods. (2). Different algorithms exhibit varying effectiveness in internet financial risk prediction. Overall, there is an improvement in prediction accuracy, time efficiency, and robustness with algorithm optimization. (3). Technological advancements also bring about technological risks [28], emphasizing the need for continuous improvement in risk anticipation and prevention.

**Table 5:** Summary of findings for literatures.

Authors	Title	Finding
Bussmann et al. [11]	Explainable AI in Fintech Risk Management	Using sophisticated machine learning models can effectively enhance the predictive accuracy of credit risk on internet financial platforms. The impact of each factor can be elucidated by employing processing methods based on Shapley values.
Cao [36]	Internet financial supervision based on machine learning and improved neural network	The adoption of an internet financial regulatory system model based on machine learning algorithms and enhanced neural network techniques yields significant improvement effects.
Chen and Jiang [7]	Internet Financial Risk Model Evaluation and Control Decision Based on Big Data	Utilizing a big data-driven internet financial risk model enables better detection of risk issues, reducing the likelihood of platform risk occurrences. Significant improvements have been achieved in areas such as customer default rates, profitability, and risk processing time.
Dong [14]	Intelligent early warning of internet financial risks based on mobile computing	The incorporation of quantitative algorithms has elevated the search efficiency of the original algorithm, enabling internet financial institutions to attain more precise risk prediction models and warning intervals.
Fan et al. [2]	Improved ML-Based Technique for Credit Card Scoring in Internet Financial Risk Control	Compared to traditional credit scoring models, machine learning algorithms are better suited to meet the data processing demands of internet financial platforms. They enhance model prediction outcomes, elevate risk control proficiency, and improve overall efficiency.
Feng and Qu [18]	Analyzing the Internet financial market risk management using data mining and deep learning methods	ACO-Optimized RBF Algorithm demonstrates superior accuracy and speed as a potential model for identifying internet financial risks.
Guang et al. [20]	Internet Financial Risk Monitoring and Evaluation Based on GABP Algorithm	GABP Algorithm Based on Simulated Annealing Optimization exhibits higher predictive accuracy and learning efficiency, achieving superior forecasting outcomes.
Han et al. [8]	Risk Analysis and Establishment of Supervision System of Internet Finance Based on Big Data Era	Compared to traditional warning models, the neural network model enhanced with data mining techniques effectively enhances the internet financial risk management by addressing uncertainty, enhancing fault tolerance, and exhibiting self-learning capabilities.
Liu et al. [25]	Analysis of Internet Financial Risk Control Model Based on Machine Learning Algorithms	Machine learning models excel in pinpointing the crucial factors that influence internet financial credit risk, thus enhancing the effectiveness of credit risk control.
Liu et al. [26]	Analysis of Internet Financial Risks Based on Deep Learning and BP Neural Network	The model based on deep learning and BP neural networks has bolstered the predictive capability of Chinese internet financial risks, effectively enhancing the security of the financial system.
Mirza et al. [19]	Safeguarding FinTech innovations with machine learning: Comparative assessment of various approaches	Machine learning techniques can automatically detect fraudulent activities in internet financial transactions. Feature selection and analysis techniques contribute to improving detection accuracy.
Noor et al. [15]	A machine learning-based FinTech cyber threat attribution framework using high-level indicators of compromise	Through the comparison of various machine learning models, it was observed that the DLNN model outperforms the other four models, demonstrating superior accuracy, recall rate, f-measure, and lower false positive rate (FPR) in characterizing network threats.
Pi et al. [28]	The Analysis of Fintech Risks in China: Based on Fuzzy Models	The fuzzy set analysis method revealed that within fintech risks, technological risk stands as one of the dominant factors influencing fintech risk.
Qi et al. [17]	Internet financial risk management and control based on improved rough set algorithm	The KNN rough set model is well-suited for handling the vague, heterogeneous, and incomplete data risks present in internet financial platforms.
Wu et al. [1]	Research on internet financial risk control based on deep learning algorithm	An internet financial risk management system based on deep learning algorithms can address issues of inadequate risk control methods and underutilization of big data. It concurrently reduces manual costs while enhancing monitoring efficiency.
Xia et al. [23]	Identifying Fintech risk through machine learning: analyzing the Q&A text of an online loan investment platform	By employing the machine learning models uncovered in this study, it becomes possible to identify textual risks posted on internet financial platforms. This capability can guide investors in their investment strategies and enhance the management of financial technology platforms.
Zang [27]	Construction of Mobile Internet Financial Risk Cautioning Framework Based on BP Neural Network	Among the various financial risk prediction frameworks, the novel BP-NN-based framework exhibits the highest accuracy in forecasting the level of MIF (Market, Interest rate, and Financial) risks.

Therefore, future research should continue to explore and expand various machine learning algorithms, particularly the application of deep learning algorithms in the field of internet financial risk. A comprehensive and sustainable risk management strategy is imperative for internet financial platform companies, investors, borrowers, regulatory authorities, and even traditional institutions like banks engaged in internet financial operations.

### 4.7 Evaluation criteria

Table 6 lists all the evaluation metrics and the formula of the metrics used in the literature for assessing internet financial risks. These metrics are employed to gauge the strengths and weaknesses of various machine learning and other methods. TP represents the number of true positive predictions, FN represents the number of false negative predictions, FP represents the number of false positive predictions, and TN represents the number of true negative predictions. ROC is commonly used to evaluate the performance of binary classifiers, where the vertical axis represents the True Positive Rate (TPR) and the horizontal axis represents the False Positive Rate (FPR). The dashed line represents the baseline, indicating the lowest standard. ROC is used on this coordinate axis to measure the accuracy of the model. The closer the ROC curve is to the upper left corner, the higher the predictive accuracy of the model. Compared to other metrics, the ROC curve can more visually display the strengths and weaknesses of different models on a graph. The Area Under Curve (AUC) refers to the area enclosed by the Receiver Operating Characteristic (ROC) curve and the x-axis. Its maximum value is 1. A larger AUC indicates a higher efficiency of the model in identifying targets [2].

**Table 6:** The evaluation metrics and formula involved in publications by year.

	2019	2020	2021	2022	2023	Total	Formula or Meaning
Accuracy rate	1	4	3	5	3	16	(TP+TN)/
							(TP+TN+FN+FP)
True positive rate(Recall)	1	2	1		2	6	TP/(TP+FN)
Precision	1	2		2		5	TP/(TP+FP)
False positive rate(FPR)	1	2			1	4	FP/(FP+FN)
ROC		2			2	4	Receiver Operating Characteristic
AUC		2			1	3	Area Under Curve
F1 score	1	2				3	2*Precision*Recall/
							(Precision+Recall)

From the perspective of the final evaluation metrics, undoubtedly, the most important evaluation metric is accuracy, which is mentioned in 16 articles. Accuracy refers to the proportion of correctly classified samples out of the total number of samples, i.e., the sum of the number of instances where the predicted value matches the actual value, divided by the total number of samples. This metric is the fundamental indicator for evaluating model performance. However, for imbalanced datasets, accuracy may not be reliable. Hence, although accuracy is widely used in literature, it is not considered the sole measure of performance.

Following that is true positive rate (Recall) which is used in 6 papers. Recall is the proportion of correctly classified positive samples out of the total number of true positive samples. Recall focuses on the statistical measure of some samples and emphasizes the correct identification of true positive samples. Then precision is used in 5 papers. Precision examines the probability of true positive samples among all predicted positive samples, indicating the confidence in correctly predicting positive samples. It measures the accuracy of positive predictions or the proportion of accurately identified positive samples. Recall focuses on how many positive instances were missed. The higher the recall, the stronger the model’s ability to distinguish positive samples. Precision, on the other hand, focuses on the proportion of predicted positives that are actually true positives. A higher precision indicates a stronger ability of the model to distinguish negative samples. Therefore, precision and recall have a trade-off relationship, each serving its purpose.

Then false positive rate (FPR) is used in 4 papers, which measures the percentage of all actual negative samples that were incorrectly classified as positive by the model. But a more comprehensive and objective evaluation metric and measurement method are the ROC curve and the AUC, which are the combined curves composed of true positive rate and false positive rate and the area under the curves, respectively. They are often used to assess the model overall, and these evaluation metrics can reduce interference from different test sets, providing a more objective measure of the model’s performance compared to individual metrics mentioned above. The ROC and AUC metrics were used in 4 and 3 articles, respectively.

F1 score is used in 3 articles. The F1 score integrates both precision and recall factors, achieving a balance between the two, ensuring both "precision" and "recall" are considered without bias. The F1 score is the harmonic mean of precision and recall, thus it simultaneously considers both the accuracy and recall of the model. However, because it is composed of the product of recall and precision, when the values of recall or precision are very small, the F1 score will also be very small. Regardless of how high one value is, if the other value is very small, the F1 score will be small as well. Therefore, it comprehensively reflects the effectiveness of the model. Using the F1 score as an evaluation metric can prevent the occurrence of extreme cases as mentioned above. Noor et al. [15] utilizes metrics such as Accuracy, Precision, Recall, F1-measure, False Positive Rate, etc., to assess and compare the effectiveness of Naïve Bayes, KNN, Decision Tree, Random Forest, and DLNN methods, thereby enabling a more comprehensive analysis and evaluation.

The results of these evaluation index indicate that higher accuracy or recall are the most intuitive indicator for assessing different methods, and it’s highly regarded by all researchers. This core metric is crucial in comparing various algorithms. The extensive and diverse set of metrics also provides us with analytical insights and frameworks for assessing the applicability of different methods in the future. Consequently, regardless of how far machine learning algorithms evolve in the future, these metrics and frameworks will continue to help us establish an effective judgment system.

## 5. Findings and discussion

The development of internet financial platforms has gone through initial rapid expansion followed by a period of gradual regulation, eventually transitioning into a stable operating phase guided by long-term goals. Given the rapid advancement of the internet and the significant role of finance in societal development, recognizing, anticipating, supervising, and managing internet financial risks have become critical topics. Utilizing techniques like machine learning to address the challenges of open internet environments and the abundance of data in financial risk prevention is both timely and necessary. In this study, we employed a systematic approach to review the internet financial risk research conducted using machine learning methods up to the present. This paper listed the machine learning models and algorithms currently used in internet finance risk management, addressing the first question posed. Future research can continue to explore areas such as research methods, data analysis, evaluation metrics, and research scope.

First and foremost, through our analysis, we have observed that whether it’s traditional machine learning algorithms, deep learning, neural networks, or other methods, all have the potential to improve prediction accuracy, surpassing traditional credit indicator calculation methods. This addresses the first question raised in this paper and also touches upon the effectiveness of machine learning methods applied to internet finance risk, addressing the second question. However, the accuracy of neural network models in predicting internet financial risks is contingent on factors such as model structure, sample data, and parameter settings [18]. There exist issues of data imbalance in the utilized datasets [23], and most algorithms exhibit certain biases in their final accuracy [27]. Hence, in the future, due to the specific requirements of the financial industry, ongoing optimization and improvement are necessary at both the algorithmic and data levels. This could involve the incorporation of new or updated algorithms more tailored to financial risks, especially algorithms suitable for extreme value research in risk identification. Although studies have developed models that are well-suited for handling fuzzy, heterogeneous, and incomplete data [17], currently, analysis of extreme cases is lacking, but financial risks or issues demand attention to extreme situations [23]. Simultaneously, in terms of data, the inherent nature of financial platforms makes obtaining timely, reliable, stable, and diverse data somewhat challenging. However, this aspect is crucial for enhancing the effectiveness of algorithms and models, given the limited quantity of research in this area at present.

Comparing different machine learning models and algorithms, the current state of affairs generally reflects that intelligent algorithms, represented by various deep learning algorithms, exhibit higher predictive accuracy compared to traditional machine learning models. They can address issues such as uncertainty, poor fault tolerance, and lack of self-learning capabilities in traditional warning models [8]. However, overall, scholars employ diverse platforms and datasets, and no study has comprehensively compared all mainstream machine learning models and algorithms. Consequently, there is no universally optimal model applicable to all platforms, addressing the third question posed in this paper.

Currently, there are multiple sources of risk in internet finance, including financial risk, legal risk, credit risk, market risk, and technological risk. Scholars primarily focus on credit risk [25] and technological risk [19]. Although some researchers have found that technological risk, ethical risk, and legal risk are the predominant factors affecting fintech risk [28], and even attempted to establish an internet finance risk control system based on deep learning algorithms [1], a considerable portion of literature still assesses machine learning algorithms from the perspective of credit risk. They evaluate whether single or multiple models can reduce expected losses [36], increase platform revenue [7], and obtain more reliable risk predictions [2, 14].

Given the characteristics of the internet finance sector, which involve short timeframes and large quantities of data [2], it is inevitable to opt for artificial intelligence risk warning and management models based on machine learning algorithms. However, the mentioned literature predominantly focuses on data within internet financial platforms or companies, without considering the influence of the external environment and other external sources or third-party data [26], which limits the generalizability of prediction results. Few studies have concentrated on machine learning identification of textual data, even though in the operational process of internet financial platforms, effective communication among users can be enhanced. Developing more timely and effective sentiment analysis algorithms for textual data could improve risk identification strategies. Thus, from this perspective, the existing internet financial risk assessment metric system should be further refined. It should incorporate external environmental data, existing credit scoring factors, third-party data, and the evaluation metrics presented in this study [18]. Establishing a more comprehensive and rational internet financial risk assessment metric system can be a potential direction for future research.

Through the analysis of evaluation metrics used in all the literature reviewed, it is evident that most studies choose accuracy, recall, and precision as metrics for evaluation and comparison of results, while fewer studies apply metrics such as ROC, AUC, F-score, and even more comprehensive and complex indicators. None of the literature covered the use of newer models and algorithms like Transformer. These observations indicate that although machine learning has been extensively applied in many fields, research in the domain of internet finance risk management remains limited. Therefore, we outline potential research directions in the "Future Research" section.

Finally, it’s evident that the majority of current applications and research on machine learning in the field of internet financial risk are conducted by Chinese scholars, using Chinese data, and considering Chinese scenarios (11 articles). Therefore, the scope and focus of research are still quite limited. With the increasing adoption of financial technology, digital currencies, big data, the Internet of Things, artificial intelligence, cloud computing, and other technologies across various countries [28], a more extensive and diverse range of research scenarios and scopes should become a mainstream in future research. This would contribute to providing a safer internet financial environment for individuals, businesses, platforms, local governments, and regulatory authorities.

## 6. Conclusion

With the gradual penetration of internet financial services in society and the maturation of machine learning algorithms, this study systematically introduces the research of machine learning models and algorithms in the field of internet financial risk. The focus is on exploring various algorithms and their characteristics used in previous studies. While, in general, machine learning enhances the accuracy of internet financial risk identification, scholars’ conclusions vary due to different approaches, and research is overly concentrated in China. Using permutations and combinations of different expressions related to "internet," "finance," and "risk" as keywords, comprehensive searches were conducted in both the Scopus and Web of Science databases, yielding 116 and 48 articles respectively. After filtering by language, document type, topic, merging, deduplication, and focusing on reading and screening content related to "machine learning," the final sample was narrowed down to 17 articles. This paper provides a comprehensive analysis of the sample literature from aspects such as annual trends, regional distribution, literature focus, fields of sciences, used models and algorithms, research findings, and evaluation metrics. Subsequently, the findings of this paper are discussed. Ultimately, it identifies research gaps and proposes future research directions in this field.

The research findings of this paper reveal that although the overall quantity is limited, the research on this topic has tripled in the past three years, with two-thirds of the studies focusing on China. Looking at the machine learning algorithms employed by scholars, a range of traditional algorithms, deep learning algorithms, and novel algorithms like neural networks have been used. The research findings consistently show that compared to traditional credit evaluation methods, machine learning models and algorithms can significantly enhance the accuracy of internet financial risk identification. However, there are noticeable differences among different algorithms, and though conclusions differ with varying datasets, generally, more recent algorithms yield higher accuracy. Additionally, scholars evaluate the effectiveness of various algorithms from aspects such as learning efficiency, recall rate, true positive rate, and more. Our study provides a comprehensive review of the current state of research involving the application of machine learning to internet financial risk. We have identified certain limitations in existing literature, such as the restrictions in research methods, the limited application of various algorithms, incomplete data analysis, exclusion of external environmental data, optimization of evaluation metrics, and over-concentration on China.

## 7. Future research

The uniqueness of this study lies in its exploration of this emerging research field, offering a comprehensive review of the application of machine learning algorithms in internet financial risk management. Overall, research on machine learning in the field of internet finance risk management is not extensive, and the findings are inconsistent. Thus, it provides innovative analytical outcomes and future research suggestions for this area. Firstly, due to scholars using different platforms, data, models, and algorithms, there is no universally accepted best model. Hence, industry practitioners can categorize discussions on different machine learning algorithms in internet finance risk management based on our research, exploring the most suitable machine learning algorithms for their own specific scenarios. Secondly, a more detailed analysis of the application considerations of deep learning models and algorithms in internet finance risk management practice is needed, starting with data acquisition to improve model efficiency. Thirdly, as mentioned earlier, the literature used in this study comes from two databases, WOS and Scopus. Expanding the literature sources while ensuring quality could be beneficial. Fourthly, future research could gradually expand its scope by merging traditional statistical analysis with machine learning methods for studying internet financial risks. Lastly, some listed companies have claimed that models based on the Transformer architecture have been applied in vertical fields such as financial risk and public security, utilizing encoders and decoders for multi-step prediction. This is also an important research direction for future identification of internet finance risks. Additionally, attention could be directed towards the impact of emerging technologies or business models like digital currencies, metaverse, and blockchain on internet financial risks.

## Supporting information

S1 File PRISMA checklist. (DOCX)

S2 File Data search result of Scopus. (CSV)

S3 File Data search result of WOS. (XLS)
