# Correction: Machine learning in internet financial risk management: A systematic literature review

**DOI:** 10.1371/journal.pone.0306364

**Published:** 2024-06-27

**Authors:** Xu Tian, ZongYi Tian, Saleh F. A. Khatib, Yan Wang

There are errors in the Funding statement. The correct Funding statement is as follows: Open Fund Project of Science and Technology Finance Key Laboratory of Hebei Province (STFCIC202213; STFCIC202102); S&T Program of Hebei (22567630H); Hebei Social Science Fund (HB22YJ026); Baoding Science and Technology Bureau science and technology plan soft science project (2340ZZ013).
